# Investigation of risk factors associated with the development of depressive symptoms in healthy subjects exposed to long‐term stress: A prospective study of the Japanese Antarctic research expedition wintering party

**DOI:** 10.1002/npr2.12479

**Published:** 2024-10-31

**Authors:** Takashi Fukunishi, Miki Ono, Kazuhiko Kasuya, Takashi Ishikawa, Mina Honyashiki, Jiro Masuya, Takeshi Inoue

**Affiliations:** ^1^ Department of Psychiatry Tokyo Medical University Shinjuku‐ku Japan; ^2^ Department of Gastrointestinal and Pediatric Surgery Tokyo Medical University Shinjuku‐ku Japan; ^3^ Department of Breast Oncology and Surgery Tokyo Medical University Shinjuku‐ku Japan

**Keywords:** depression, Japanese Antarctic research expedition (JARE), neuroticism, stress, subthreshold depression

## Abstract

**Background:**

Stressors induce depression together with parenting experienced in childhood, personality traits, and sleep. In this study, we investigated factors associated with the development of depression in a long‐term stressful environment, namely, the Antarctic Research Expedition wintering party, by comparing 2 groups, the depression and nondepression groups.

**Methods:**

A self‐administered questionnaire was used to survey 91 members of the Japanese Antarctic Research Expedition who spent winters in the Antarctic base. Psychological evaluations of depression, anxiety, and sleep were performed using a questionnaire every 3 months during the participants’ stay in Antarctica. The primary endpoint was the occurrence of minor or major depression, as evaluated by the PHQ‐9 score.

**Results:**

Participants with a PHQ‐9 score of 5 or more during their stay in Antarctica were defined as the depression group (25 subjects), and participants with a PHQ score of 4 or less were defined as the nondepression group (43 subjects). Compared with the nondepression group, the depression group had significantly higher scores for predeparture PHQ‐9, state and trait anxiety, sleep disturbance, and neuroticism. Multivariable logistic regression analyses showed that higher predeparture scores of subthreshold depressive symptoms and neuroticism were found to be significant predictors of the occurrence of depression during their stay in Antarctica.

**Conclusions:**

This study prospectively showed that subthreshold depressive symptoms and neuroticism, which were suggested as risk factors in previous studies, were confirmed to be risk factors for depression. The results of our study are expected to contribute to the understanding of depression in harsh environments.

## Abstract 2

This study investigated risk factors associated with the development of depression in the Antarctic Research Expedition wintering party. This study prospectively confirmed that subthreshold depressive symptoms and neuroticism are risk factors for depression.

## Graphical abstract

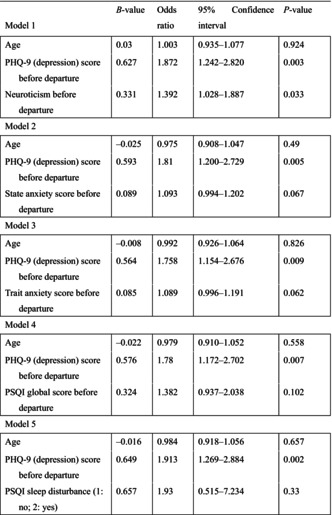

## 1 INTRODUCTION

Research in Antarctica is valuable not only for basic research, but also for medical research, and has been conducted actively for many years despite the challenging environment. In addition, research in Antarctica provides valuable information for space exploration, as Antarctica is recognized as the closest test site on Earth to space bases and is referred to as a space analog.^1^ At present, there are 20 countries, including Japan, that have winter research stations in Antarctica. Under the Antarctic Treaty, which calls for all nations to cooperate and use Antarctica in a peaceful manner, each country conducts research in atmospheric science, meteorology, and other fields in Antarctica.^2^ Antarctica is a harsh environment for humans, with extreme cold and large seasonal changes in daylight hours. In addition to the above difficulties, Antarctic winter research parties often experience major changes in mood and sleep during their extended stay owing to isolation for more than a year, limited private space, and monotony of life.^3^

Previous studies on sleep disturbances and depression in Antarctic winter research party members have been conducted, and reported that prolonged isolation in Antarctic winter research bases could cause sleep disturbances, depression, and other psychiatric symptoms. Psychiatric disorders evaluated by Diagnostic and Statistical Manual of Mental Disorders‐IV occurred in 5.2% of the members during their extended stay in Antarctica. Mood disorders were the most common, accounting for 30.2% of all cases of psychiatric disorders, followed by adjustment disorders (27.9%), sleep‐associated disorders (20.9%), personality disorders (11.6%), and substance‐associated disorders (9.3%).^4^ Having a psychiatric disorder may interfere with subsequent work at the Antarctic base. There have also been cases reported previously of mental illness leading to suicide during or after Antarctic expeditions.^5^ As a result, psychological screening programs have been developed for potential Antarctic research party candidates.^6^ Screening not only identifies candidates with a history of mental illness or current psychiatric symptoms but also has life‐saving implications by selecting candidates with high task performance and emotional stability.^3^ Among the mental health interventions, predicting depression and depressive symptoms is of paramount importance in terms of suicide prevention for Antarctic expedition personnel.^7^

Depression is a common psychiatric disorder with a lifetime prevalence rate of 15%–18%, and in recent years, its disease burden has been worsening worldwide.^8^, ^9^ Furthermore, absenteeism and reduced productivity owing to depression also cause substantial economic losses,^10^ making elucidation of the etiology of depression and improvement of its prevention and treatment crucial, not only in Japan but also worldwide. It is now known that various risk factors are intricately involved in the development of depression. Genetics, sex differences, childhood abuse, childhood parenting environment, various personality traits, such as neuroticism and trait anxiety, insomnia, and stressful life events have been suggested to play complex roles in the development of depression or the appearance of depressive symptoms.^7^, ^8^, ^11^, ^12^, ^13^, ^14^, ^15^, ^16^, ^17^, ^18^ The most established line of evidence for the development of depression is that depression is more likely to occur in highly neurotic individuals when they are exposed to stressful life events.^13^, ^19^ Therefore, it is possible that neuroticism together with other factors may be involved as risk factors for the development of depression or depressive symptoms in individuals on Antarctic expeditions, but this has not been fully investigated to date.

Personal characteristics suitable for Antarctic exploration have been investigated previously. In particular, 3 personal characteristics, namely, emotional stability, task management ability, and social compatibility, have been shown to predict adaptability in Antarctic exploration.^3^, ^20^ Low neuroticism, low depressive symptoms, and emotional stability were found to predict optimum performance and adaptability in Antarctic expeditions.^3^, ^6^ High neuroticism and military status were identified as risk factors predicting the occurrence of depressive symptoms in Antarctic expeditions.^6^ However, because the assessment of depressive symptoms was neither performed using a standard psychiatric assessment nor a screening test for depression,^6^ this previous study had the major limitation of not being a standard psychiatric investigation. Therefore, to our knowledge, there have been no prospective studies to date on the exacerbation of depressive symptoms or the occurrence of depressive episodes during Antarctic expeditions.

In the present study, we hypothesized that risk factors for the development of depression during a 1‐year Antarctic expedition were the same as those found in previous studies, and investigated risk factors for the development of mild to major depression in healthy individuals during a 1‐year Antarctic research expedition of the Japanese Antarctic Research Expedition (JARE). Risk factors including parenting experienced in childhood,^12^, ^15^ neuroticism,^12^, ^13^, ^15^ state and trait anxiety,^12^, ^18^ and sleep disturbances^11^ were investigated. To confirm our hypothesis, we conducted a prospective study of risk factors for the development of depressive episodes among members of the JARE, in which uniform stress is applied to a large number of healthy individuals, who are followed for one year to analyze psychological changes in Antarctic research expedition members who traveled from Japan to the Antarctic and experienced an isolating winter, while overcoming harsh conditions. Factors associated with the development of depressive episodes in a long‐term stressful environment of the Antarctic research expedition were identified by multivariable logistic regression analysis, comparing the depression group and nondepression group as a dependent variable.

## 2 METHODS

### 2.1 Participants

A total of 91 members of the JARE were asked to participate in the survey using a self‐administered questionnaire between October 2017 and December 2020, and provided written informed consent. Of those, 7 were excluded owing to several missing values, and 16 were excluded as they had a Patient Health Questionnaire‐9 (PHQ‐9) score of 5 or higher, exceeding the threshold for mild depression, before their travel to the Antarctic. Finally, 68 members of the Antarctic winter research party were included in the analysis. It should be noted that only basically healthy people were selected for the Antarctic winter research party. Participants were informed that their participation in this study was voluntary, that they would not be disadvantaged if they did not agree to participate, and that their data would be strictly controlled and their personal information would be carefully managed. This study was conducted in accordance with the Declaration of Helsinki (revised in Fortaleza in 2013), and was approved by the Medical Ethics Review Committee of Tokyo Medical University (study approval number: SH3712) and the Ethics Review Committee of the National Institute of Polar Research.

### 2.2 Questionnaires

Questionnaires were administered to the participants before they left for Antarctica (October) and every 3 months (March, June, September, and December) during their stay in Antarctica. Demographic information and results of the Parental Bonding Instrument (PBI) were collected only before the travel.

#### 2.2.1 Parental bonding instrument (PBI)

The PBI is a subjective rating scale of parental attitudes experienced in childhood from the participants' perspective.^21^ In the PBI, a higher “care” score indicates a higher tendency of parents to provide emotional warmth, empathy, and closeness (lower tendency for indifference and neglect), and a higher “overprotection” score indicates a higher tendency of parents to perform intrusion, excessive contact, and infantilization (lower tendency of allowing independence). Long‐term stability of the PBI over a 20‐year period was reported.^22^ A Japanese version of the PBI has been developed by Kitamura and Suzuki,^23^ and its validity and reliability have been confirmed. Subscores for maternal and paternal care and overprotection were used in the analysis.

#### 2.2.2 Patient health questionnaire‐9 (PHQ‐9)

The PHQ‐9 is a self‐administered depression rating scale consisting of 9 items, the total score of which indicates the severity of depressive symptoms.^24^ The Japanese version of the validated PHQ‐9 was used in this study.^25^ The total score (range: 0–27 points) was used for analysis. A total PHQ‐9 score of 0 to 4 was defined as “no depression,” a score of 5 to 9 as “mild depression,” and a score of 10 or higher as “major depression.”^26^, ^27^ Therefore, a total PHQ‐9 score of 5 or higher was regarded as “depression” in this study.

#### 2.2.3 State–trait anxiety inventory‐Y (STAI‐Y)

The STAI‐Y is a 40‐question questionnaire on anxiety symptoms, consisting of 20 questions each on state anxiety and trait anxiety.^28^ State anxiety assesses anxiety symptoms that the subject is feeling at that moment, whereas trait anxiety assesses anxiety symptoms that are usually or generically felt, namely, a relatively stable tendency to react to anxiety‐evoking events or an anxiety‐associated personal trait. In the present study, both state anxiety and trait anxiety were used for analysis. The Japanese version of the questionnaire was used.^29^

#### 2.2.4 Eysenck personality questionnaire‐revised (EPQ‐R) neuroticism subscale

Neuroticism was measured using a subscale of the self‐administered EPQ‐R shortened Japanese version.^30^, ^31^ This scale is a yes‐no questionnaire consisting of 12 items (no = 0; yes = 1). High scores indicate that subjects are more likely to have feelings of anxiety, worry, and irritability; the validity and reliability of the Japanese version of the EPQ‐R shortened version were confirmed in our previous study.^31^ In the present study, the total score of the 12 items was used as the score of neuroticism.

#### 2.2.5 Pittsburgh sleep quality index (PSQI)

The PSQI is a self‐administered questionnaire that assesses sleep disturbance in subjects.^32^ The PSQI consists of 7 subscales (C1: sleep quality; C2: sleep latency; C3: sleep duration; C4: habitual sleep efficiency; C5: sleep disturbance; C6: sleep medication use; C7: daytime dysfunction). Each subscale is rated on a scale of 0 to 3, thus the global score (total score) ranged from 0 to 21, reflecting the degree of sleep disturbance; a global score of 6 or more was defined as having sleep disturbance.^32^ The validated Japanese version of the PSQI (PSQI‐J) was used in this study.^33^

#### 2.2.6 Demographic information

Demographic information included age, sex, years of education, marital status, past history of psychiatric treatment, current psychiatric treatment, physical disease, and subjective social status.

### 2.3 Statistical analysis

The depression group (25 participants) was defined as those who scored 5 or higher on the PHQ‐9 at least once during the 3‐month post‐travel evaluation, and the nondepression group (43 participants) was defined as those who scored 4 or lower on the PHQ‐9 continuously. First, demographic information and scores on the PBI, PHQ‐9, STAI‐Y, and EPQ‐R for the depression and nondepression groups were compared using the *t*‐test and the Fisher exact probability or χ^2^ test (SPSS 28.0, IBM, Armonk, NY, USA). Next, multivariable logistic regression analysis was conducted using predeparture neuroticism, state anxiety, trait anxiety, degree of sleep disturbance, presence of sleep disturbance, and depressive symptoms as independent variables, and the distinction between the depression group and the nondepression group as the dependent variable. Age and the degree of predeparture depressive symptoms were controlled for. As there were only 25 individuals in the depression group, which was a smaller number than that in the nondepression group, there were only 3 independent variables that could be entered into the multivariable logistic regression analysis.^34^ A *p*‐value of less than 0.05 was set as the statistical significance level.

## 3 RESULTS

Twenty‐five subjects had a PHQ‐9 score of 5 or higher (i.e., mild or major depression) at least once during each of the assessments every 3 months during the Antarctic expedition, and 43 subjects had a PHQ‐9 score of 4 or lower during their entire stay in Antarctica. As only 8 subjects experienced major depression (PHQ‐9 score of 10 or higher) during the Antarctic expedition, subjects with mild or major depression with a PHQ‐9 score of 5 or higher at least once during their stay in Antarctica were defined as the depression group. The time course of changes in PHQ‐9 total scores in the nondepression and depression groups is shown in Figure S1.

Table 1 shows the results of the comparison of demographic information and predeparture PBI, PHQ‐9, STAI‐Y (state and trait anxiety), and EPQ‐R neuroticism scores between the depression group and the non‐depression group. Demographic information and PBI subscales were not significantly different between the 2 groups. Depressive symptoms (PHQ‐9), STAI‐Y state and trait anxiety, EPQ‐R neuroticism, PSQI global score (degree of sleep disturbance), and presence of sleep disturbance (PSQI global score ≥6) before departure to Antarctica were significantly higher in the depression group than in the nondepression group.

**TABLE 1:** Characteristics and questionnaire scores of the participants.

	Nondepression group (*n* = 43)	Depression group (*n* = 25)	*p*‐value	Statistical test
Age (years)	40.1 ± 9.1	38.6 ± 7.5	ns	a
Sex (male: female)	41: 2	22: 3	ns	b
Years of education	15.5 ± 3.1	16.0 ± 2.6	ns	a
Marital status (unmarried: married)	16: 26	9: 16	ns	c
Subjective social status (1 [lowest] to 10 [highest])	5.8 ± 1.3	5.7 ± 1.2	ns	a
Physical disease (no: yes)	26: 0	17: 2	ns	b
Past psychiatric treatment history (no: yes)	41: 1	25: 0	ns	b
Current psychiatric treatment (no: yes)	42: 0	25: 0	ns	b
Before departure				
PBI paternal care	24.6 ± 8.2	24.6 ± 6.1	ns	a
PBI paternal overprotection	8.0 ± 5.2	10.0 ± 5.6	ns	a
PBI maternal care	29.2 ± 5.8	27.0 ± 5.6	ns	a
PBI maternal overprotection	8.3 ± 6.5	10.6 ± 5.9	ns	a
PHQ‐9 score (depression)	0.7 ± 1.1	2.2 ± 1.6	<0.001	a
STAI‐Y state anxiety score	35.6 ± 6.9	40.5 ± 6.4	0.005	a
STAI‐Y trait anxiety score	35.3 ± 6.9	41.4 ± 7.1	<0.001	a
EPQ‐R neuroticism score	1.7 ± 1.7	3.3 ± 2.2	0.002	a
PSQI global score (sleep disturbance)	3.7 ± 1.7	5.0 ± 1.7	0.003	a
Sleep disturbance (no: yes)	36: 7	15: 10	0.042	c

*Note*: Data are shown as the mean ± standard deviation or numbers.Statistical test: (a) *t*‐test, (b) Fisher's exact test, (c) χ^2^‐test.

Abbreviations: EPQ‐R, Eysenck Personality Questionnaire‐Revised; ns: not significant; PBI, Parental Bonding Instrument; PHQ‐9, Patient Health Questionnaire‐9; PSQI, Pittsburgh Sleep Quality Index; STAI‐Y, State–Trait Anxiety Inventory‐Y.

Table 2 shows the results of the multivariable logistic regression analysis with the distinction between the depression group and the nondepression group as the dependent variable. The results showed that the severity of depressive symptoms before departure was a predictor of depression. Age was not associated with depression. When controlling for age and the severity of predeparture depressive symptoms, only predeparture neuroticism was a significant predictor of depression during the Antarctic expedition. State anxiety, trait anxiety, degree of sleep disturbance, and presence of sleep disturbance before departure were not significant predictors of depression during the Antarctic expedition. As noted in the Methods section, as predeparture PHQ‐9 scores of only 4 or less were included, predeparture subthreshold depressive symptoms of the participants were very mild and within the asymptomatic range.

**TABLE 2:** Multivariable logistic regression analyses by the forced entry method of the diagnosis of nondepression (*n* = 43) and depression (*n* = 25) as dependent variables.

	*B*‐value	Odds ratio	95% confidence interval	*p*‐value
Model 1				
Age	0.03	1.003	0.935–1.077	0.924
PHQ‐9 (depression) score before departure	0.627	1.872	1.242–2.820	0.003
Neuroticism before departure	0.331	1.392	1.028–1.887	0.033
Model 2				
Age	−0.025	0.975	0.908–1.047	0.49
PHQ‐9 (depression) score before departure	0.593	1.81	1.200–2.729	0.005
State anxiety score before departure	0.089	1.093	0.994–1.202	0.067
Model 3				
Age	−0.008	0.992	0.926–1.064	0.826
PHQ‐9 (depression) score before departure	0.564	1.758	1.154–2.676	0.009
Trait anxiety score before departure	0.085	1.089	0.996–1.191	0.062
Model 4				
Age	−0.022	0.979	0.910–1.052	0.558
PHQ‐9 (depression) score before departure	0.576	1.78	1.172–2.702	0.007
PSQI global score before departure	0.324	1.382	0.937–2.038	0.102
Model 5				
Age	−0.016	0.984	0.918–1.056	0.657
PHQ‐9 (depression) score before departure	0.649	1.913	1.269–2.884	0.002
PSQI sleep disturbance (1: no; 2: yes)	0.657	1.93	0.515–7.234	0.33

Abbreviations: B, partial regression coefficient; PHQ‐9, Patient Health Questionnaire‐9; PSQI, Pittsburgh Sleep Quality Index.

## 4 DISCUSSION

The results of the present study comparing the depression and nondepression groups of research members of the JARE showed that only subthreshold depressive symptoms and neuroticism before departure to the Antarctica were significant predictors of mild or major depression. Previous studies have reported that individuals with subthreshold depressive symptoms and high neuroticism are more likely to develop depression when they are exposed to a highly stressful life event.^13^, ^19^, ^35^, ^36^, ^37^ In the present study, the life event of staying one year in Antarctica, which is an unusual and stressful life event (defined in previous studies as serious difficulties at work),^13^ and neuroticism may have influenced the onset of depression, as the findings of previous studies demonstrated that serious difficulties at work and neuroticism influence the onset of depression. Furthermore, the results of this study suggest that the mechanism of depression in community residents and Antarctic research party members is similar. Our study is the first to our knowledge to identify risk factors for the development of depression in members of the JARE performing research in Antarctica.

High anxiety traits contribute to the development of depression by increasing an individual's vulnerability to stress.^18^, ^38^ However, predeparture state and trait anxiety were not significant predictors of depression in the multivariable analysis of the present study. Although the neuroticism subscale of the EPQ‐R includes items associated primarily with anxiety, nervousness, and worry that overlap with the STAI‐Y state and trait anxiety items, the neuroticism subscale also includes items associated with feeling miserable, irritable, fed‐up, easily hurt, lonely, and guilty, which were not included in the STAI‐Y.^28^, ^30^ Thus, it is possible that items of neuroticism not shared with state and trait anxiety contributed to the occurrence of depression in subjects of the present study. As all of the above neuroticism items are characteristics associated with the emergence and worsening of interpersonal conflicts, it is possible that neuroticism contributes to the conflicts that arise in human relationships in long‐term isolation and in the confined space of the Antarctic base, leading to increased stress and the onset of depression.^13^ Furthermore, anticipatory anxiety may not be a significant predictor of depression in this study, because the members of the JARE chose this difficult assignment on their own, and it was an anticipated stress. This may explain why state and trait anxiety were not a predictor of depression.

Subthreshold depression is also known to have a high prevalence, a significant impact on patients’ quality of life, and economic losses.^35^ Several previous studies have reported that subthreshold depression is a risk factor for depression.^35^, ^37^, ^39^ In the present study, a population with a PHQ‐9 score of 0 to 4 before departure to Antarctica, which indicates the absence of depressive symptoms, was analyzed. The results of this study then showed that under the stress of the extreme conditions of an Antarctic winter expedition, higher depressive symptoms, even in the almost asymptomatic range of depressive symptoms, that is, very low‐level subthreshold depressive symptoms, are predictive of depression. A possible pathogenic mechanism is that stress added to subthreshold depressive symptoms leads to the onset of depression. Depressive symptom scores on the rating scale are continuous in populations with depression,^40^ and the degree of depressive symptoms may form a spectrum in community residents (our unpublished data). Therefore, it is easy to understand that depressive symptoms are increased by stress and become more severe, resulting in depression that exceeds the threshold level.

Although exposing people to experimental confinement conditions is ethically not permitted, the Antarctic wintering research party is a valuable research population that enables us to investigate the effects of a consensually exposed isolation environment on mental health. Findings from Antarctic wintering research parties can be applied to future space exploration or work involving long‐term isolation.^1^ Furthermore, when selecting members for the Antarctic winter research party, psychological screening to select candidates who are less likely to experience a deterioration in mental health is necessary to protect the mental health of the members. In addition to psychologically and physically healthy candidates, studies in the U.S. have indicated low neuroticism, low depressive symptoms, emotional stability, age of older than 30 years, low need for social support or order, and tolerance of the lack of achievement as characteristics suitable for Antarctic wintering research party members.^3^ The low levels of subthreshold depression and low neuroticism tendency identified in this study as characteristics of subjects less likely to develop depression are partially consistent with U.S. recommendations. To prevent the development of depression in Antarctic winter research members, it would be useful to add low subthreshold depression and low neuroticism tendencies to the member screening inventory. The above lines of evidence suggest that it is necessary to support candidates, such as those with specific needs or risk factors for depression, with care and warmth. Furthermore, these characteristics could also be used for the selection of personnel required to perform missions in other extreme conditions, such as space exploration and deep‐sea exploration. It is expected that the results of this study will be useful for the selection of future Antarctic research expedition members, and will help them to safely perform their research and other missions under extreme conditions during the Antarctic winter.

## 5 LIMITATIONS

There are 3 possible limitations to this study. First, the members of the JARE who stayed one year in Antarctica are a group of healthy individuals, so the results cannot be applied to the general population. Second, the primary endpoint was depression evaluated by a self‐administered questionnaire, as no psychiatrists participated in the Antarctic expedition. Therefore, the diagnosis may have differed if the participants were evaluated by psychiatrists. Third, the power of the statistics may not be sufficient, owing to the small sample size. As there were only 25 individuals in the depression group, only 3 independent variables could be entered into the multivariable logistic regression analysis.^34^ Therefore, confounding factors were not sufficiently controlled for in the present study. In future studies, the present findings should be confirmed using a larger sample size to sufficiently control for various confounding factors.

## 6 CONCLUSIONS

In this study, we analyzed the risk factors for the occurrence of depression in a relatively homogeneously stressed population of individuals from the Antarctic winter research party in a prospective manner. Subthreshold depressive symptoms and neuroticism, which have been implicated in the development of depression in community residents, were also identified as risk factors for the occurrence of depression in this population. On the other hand, anxiety symptoms (trait anxiety and state anxiety) and sleep disturbances, which have also been suggested to be associated with depression in common situations, were not associated with the occurrence of depression in this population. Therefore, it is suggested that individuals without subthreshold depressive symptoms and those with low neuroticism should be selected as research members of the JARE to enable safe conduction of the research.

## AUTHOR CONTRIBUTIONS

All authors contributed to the conception and design of the study, and to the acquisition, analysis, and interpretation of the data. All authors reviewed the manuscript draft and revised it critically for intellectual content. All authors approved the final version of the manuscript to be published. All authors agreed to be accountable for all aspects of the work, and to ensure that questions associated with the accuracy or integrity of any part of the work are appropriately investigated and resolved.

## FUNDING INFORMATION

This work was supported in part by a Grant‐in‐Aid for Scientific Research (no. 21K07510 to T. Inoue) from Japan Society for the Promotion of Science (JSPS) and by Japan Agency for Medical Research and Development (AMED) (grant number JP23rea522113).

## CONFLICT OF INTEREST STATEMENT

Jiro Masuya has received personal fees from Otsuka Pharmaceutical, Eli Lilly, Astellas, and Meiji Yasuda Mental Health Foundation, as well as grants from Pfizer. Takeshi Inoue has received personal fees from Mochida Pharmaceutical, Takeda Pharmaceutical, Janssen Pharmaceutical, Novartis Pharma, MSD, Yoshitomiyakuhin, Nipro, Kyowa Pharmaceutical Industry, Viatris, Lundbeck, Boehringer Ingelheim, Ono Pharmaceutical, and Meiji Seika Pharma; grants from Daiichi Sankyo, and Tsumura; and grants and personal fees from Shionogi, Otsuka Pharmaceutical, Sumitomo Pharma, Mitsubishi Tanabe Pharma, and Eisai; and is a member of the advisory boards of Luye, Shionogi, GlaxoSmithKline, Viatris, and Otsuka Pharmaceutical. The other authors do not have any actual or potential conflicts of interest to declare.

## ETHICS STATEMENT

Approval of the Research Protocol by an Institutional Reviewer Board: This study was conducted in accordance with the Declaration of Helsinki, and was approved by the Medical Ethics Review Committee of Tokyo Medical University (study approval no.: SH3712) and the Ethics Review Committee of the National Institute of Polar Research.

Informed Consent: All participants received an explanation of the purpose and method of the study, and provided prior written informed consent to participate in the study.

Registry and Registration No. of the Study/Trial: N/A.

Animal Studies: N/A.

## Supporting information


Figure S1.

## Data Availability

The institutional review board of Tokyo Medical University has restricted data sharing because the data contain potentially identifying or sensitive participant information. Please contact the institutional review board of Tokyo Medical University with data requests. Upon request, the board will decide whether to share the data.
