# Supplementary material for: Investigation of risk factors associated with the development of depressive symptoms in healthy subjects exposed to long‐term stress: A prospective study of the Japanese Antarctic research expedition wintering party
Source: Neuropsychopharmacol Rep. 2024 Oct 31;44(4):821–8. doi: 10.1002/npr2.12479 (PMC11609754; doi:10.1002/npr2.12479)
Supplement: Supplementary file 1 — Figure S1. [file NPR2-44-821-s001.docx]

**Supplementary Figure 1**

**Supplementary Figure 1. Time course of changes in PHQ-9 total scores in the nondepression (A) and depression (B) groups.** PHQ-9 scores were assessed 5 times; i.e., before departure to Antarctica, and in March, June, September, and December during the participants’ stay in Antarctica.
